# Clinical Outcomes of Radial Versus Femoral Access for Percutaneous Coronary Intervention in ST-Segment Elevation Myocardial Infarction: A Retrospective Study From Southern Puerto Rico

**DOI:** 10.7759/cureus.117573

**Published:** 2026-10-07

**Authors:** Yomara Huertas Gomez, Luis Colon Gonzalez, Angel Ramos, Carla Hernandez, Alejandra Rivera, Rafael Rivera-Berrios, Eduardo Colon Melendez

**Affiliations:** 1 Internal Medicine, Centro Médico Episcopal San Lucas, Ponce, PRI; 2 Cardiology, Centro Médico Episcopal San Lucas, Ponce, PRI; 3 Medical School, Ponce Health Sciences University, Ponce, PRI; 4 Interventional Cardiology, Centro Médico Episcopal San Lucas, Ponce, PRI; 5 Internal Medicine, Hospital Episcopal San Lucas, Ponce, PRI

**Keywords:** femoral access, primary percutaneous coronary intervention (pci), puerto rican population, radial access, st-segment elevation myocardial infarction (stemi)

## Abstract

Background and aim

Patients with ST-segment elevation myocardial infarction (STEMI) frequently undergo percutaneous coronary intervention (PCI) using either radial or femoral arterial access. Prior studies have shown potential benefits of radial access; however, data are limited regarding these approaches in patients from Puerto Rico. This study aimed to compare bleeding, vascular complications, and 30-day all-cause mortality between radial and femoral access for PCI in patients with STEMI in southern Puerto Rico.

Methods

We conducted a cross-sectional review of medical records of patients aged 21-90 years admitted with STEMI at Centro Médico Episcopal San Lucas in southern Puerto Rico from 2021 to 2023. We excluded patients with non-ST-segment elevation myocardial infarction or unstable angina. A total of 311 patients were identified and included in the study. Patients were stratified according to radial or femoral access on the first attempt. Outcomes included 30-day all-cause mortality, pseudoaneurysm, hematoma, and bleeding after thrombolysis in myocardial infarction. All statistical analyses were conducted using RStudio (Boston, MA: Posit, PBC).

Results

Among the study subjects, 90 patients had first-attempt radial access, while 221 patients had first-attempt femoral access. There were no major bleeding events in any of the groups. Minor bleeding events were reported for one out of 90 patients in the radial group compared to 21 patients out of 221 in the femoral group. This difference was statistically significant (p=0.01791). Pseudoaneurysm did not occur in the radial group, while seven occurred in the femoral group; however, this difference was not statistically significant. No hematoma was seen in any patient in the first-attempt radial access group, compared with 33 patients with hematoma in the femoral access group (p=0.001). Mortality within 30 days from all causes occurred in one of 90 patients in the radial group compared with 26 of 221 patients in the femoral group. The 30-day survival rate was 98.9% in the radial group and 88.2% in the femoral group, representing a statistically significant difference (p=0.0012).

Conclusion

Among patients with STEMI undergoing PCI in southern Puerto Rico, first-attempt radial access was associated with lower rates of minor bleeding, hematoma, and 30-day all-cause mortality compared with first-attempt femoral access. The results suggest potential clinical advantages of radial access when clinically appropriate. However, since the current study is of an observational design, it is not reasonable to claim a cause-and-effect relationship. Larger studies that control for clinical factors are needed.

## Introduction

Percutaneous coronary intervention (PCI) is a non-surgical, invasive procedure with the goal of relieving narrowing or occlusion of the coronary artery and improving blood supply to ischemic tissue in patients diagnosed with ST-segment elevation myocardial infarction (STEMI), and it can be performed through either the radial or femoral artery [1]. Despite the common goal of rapid reperfusion, both approaches are associated with several complications [2,3]. Several studies have compared the clinical results of radial and femoral access for percutaneous coronary intervention (PCI) [2-8]. Although some studies have found no significant differences in clinical outcomes between the two approaches [5], current evidence supports radial access due to its association with decreased access-site bleeding, vascular complications, and mortality, especially among high-risk patients with ST-segment elevation myocardial infarction (STEMI) [1-4,6]. Compared to femoral access, radial access has lower rates of major access-site bleeding, in-hospital mortality, and other vascular complications, according to more recent national data [1,6]. However, there is still a dearth of information assessing these results, particularly among Puerto Rican patients. Therefore, we hypothesized that, in comparison to femoral access, radial access for PCI would be associated with less bleeding, vascular complications, and mortality among patients with STEMI receiving treatment in southern Puerto Rico.

## Materials and methods

We conducted a retrospective cross-sectional review of the medical records of patients aged 21-90 years who were admitted with ST-segment elevation myocardial infarction (STEMI) and underwent percutaneous coronary intervention (PCI) at Centro Médico Episcopal San Lucas, an urban hospital that serves the southern region of Puerto Rico, between 2021 and 2023. Those with non-ST-elevation myocardial infarction or unstable angina were excluded. A total of 311 patients were included and classified according to the vascular access site used at the initial access attempt (radial, n=90; femoral, n=221). No crossover between radial and femoral access sites occurred; therefore, the initial access site corresponded to the final successful access site for all patients. The demographic characteristics of the study included the year of admission, age, sex, and body mass index (BMI). The list of comorbidities comprised diabetes mellitus, obesity, hypertension, coronary artery disease, heart failure, thrombocytopenia, anemia, chronic kidney disease, acute kidney injury, end-stage renal disease, and chronic obstructive pulmonary disease. The variables related to the procedural aspects of PCI included the vascular access site, fluoroscopy time, contrast type and volume, estimated blood loss, catheter size, vascular closure device, duration of the procedure, and length of hospital stay. The outcomes studied involved the Thrombolysis in Myocardial Infarction (TIMI) classification of hemorrhage, hematoma, pseudoaneurysm, need for a blood transfusion after the PCI procedure, overall major complications, and 30-day overall mortality. According to the TIMI bleeding classification, major bleeding was defined as intracranial hemorrhage or clinically overt bleeding associated with a decrease in hemoglobin of ≥5 g/dL. Minor bleeding was defined as clinically overt bleeding associated with a decrease in hemoglobin of 3 to <5 g/dL, while minimal bleeding was defined as clinically overt bleeding associated with a decrease in hemoglobin of <3 g/dL [9]. Statistical analysis was conducted using RStudio. Descriptive statistics were calculated for baseline characteristics and clinical outcomes. Continuous variables were summarized using means and standard deviations. Procedure duration was compared between the radial and femoral access groups using an independent-samples t-test. Categorical variables were expressed as frequencies and percentages and compared using Pearson’s chi-square test or Fisher’s exact test, as appropriate based on expected cell counts. For 30-day all-cause mortality, Fisher’s exact test was used because of the small number of events in some cells. An odds ratio (OR) with a corresponding 95% confidence interval (CI) was calculated to quantify the association between vascular access site and 30-day all-cause mortality, with radial access serving as the reference group. A two-sided p<0.05 was considered statistically significant. Of the 311 patients included in the study, 274 (88.1%) had complete data for all evaluated variables, whereas 37 (11.9%) had incomplete documentation for one or more variables. Missing data identified in the statistical dataset included BMI (8/311, 2.6%), contrast type (16/311, 5.1%), hematoma (2/311, 0.6%), pseudoaneurysm (4/311, 1.3%), and vascular surgery consultation (1/311, 0.3%). Missingness was assessed separately according to vascular access group. No imputation was performed. Analyses were conducted using available observations for each variable; therefore, denominators could vary according to data availability.

## Results

A total of 311 patients with ST-segment elevation myocardial infarction undergoing percutaneous coronary intervention were included in the study. Of these, 90 patients (28.9%) underwent first-attempt radial access, and 221 patients (71.1%) underwent first-attempt femoral access. Missingness was generally low at the individual-variable level. Missing BMI data occurred in 2.2% of the radial group and 2.7% of the femoral group; contrast type was missing in 2.2% and 6.3%, respectively. Missing outcome data were confined to the femoral group for hematoma (0.9%) and pseudoaneurysm (1.8%), while vascular surgery consultation status was missing in 0.5% of the femoral group. A total of 221 patients (71.1%) were male, and 90 patients (28.9%) were female, and there was no significant difference in the sex distribution between the two groups (p=0.703) (Figure 1). There was no difference in the age distribution between groups (p=0.051). Baseline demographic characteristics and comorbidities according to vascular access site are summarized in Table 1.

**Figure 1:**
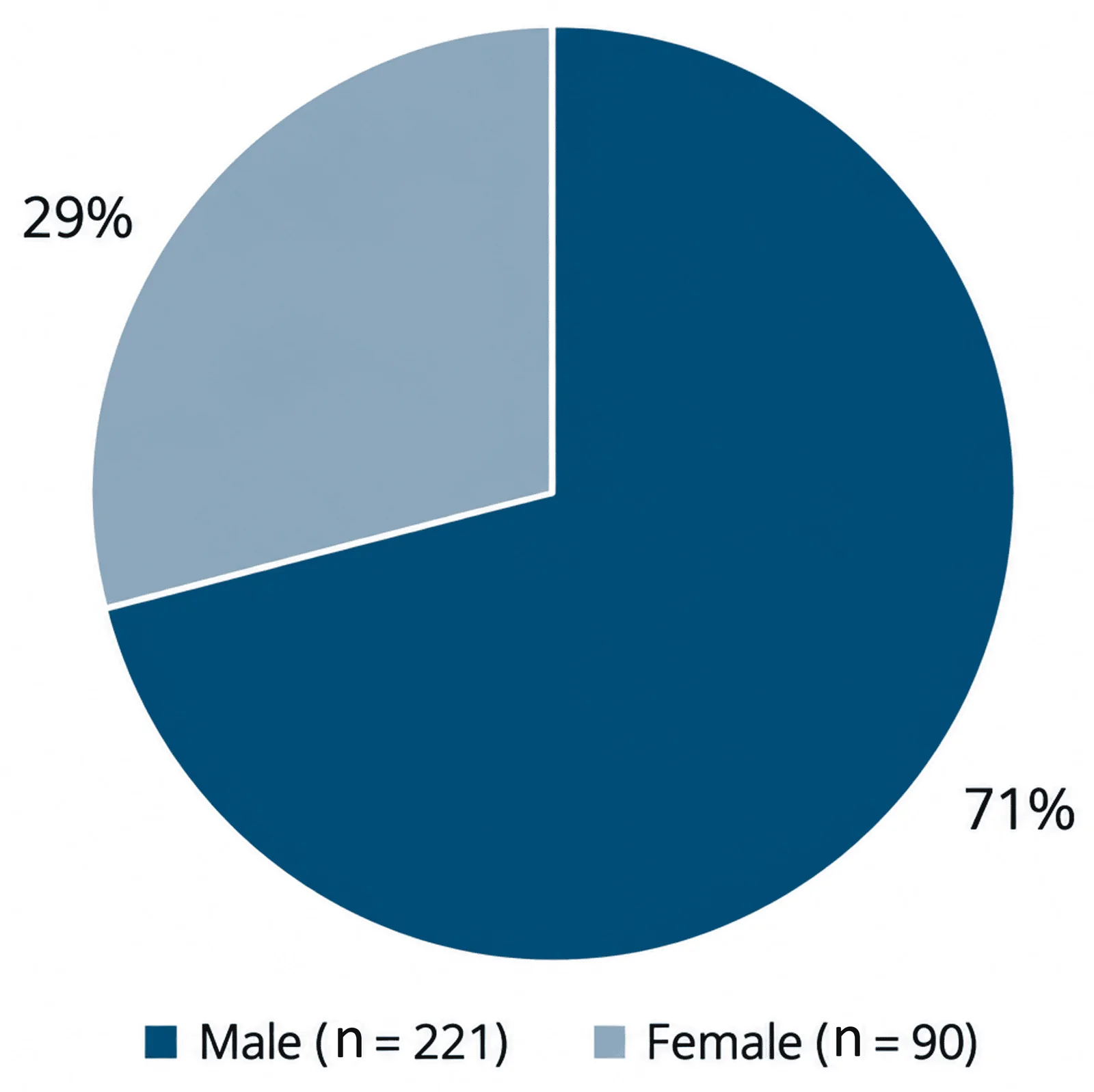
Gender distribution among the study population (N=311). Among the 311 patients included in the study, 221 (71.1%) were male and 90 (28.9%) were female. There was no statistically significant difference in sex distribution between the radial and femoral access groups (χ²=0.15, p=0.703). Statistical analysis was performed using Pearson's chi-square test.

**Table 1:** Baseline demographic and clinical characteristics according to vascular access site. COPD: chronic obstructive pulmonary disease

Characteristics	Radial access (n=90)	Femoral access (n=221)	Overall (N=311)	p-Value
Sex, n (%)				
Male	67 (74.4)	154 (69.7)	221 (71.1)	0.703
Female	23 (25.6)	67 (30.3)	90 (28.9)	
Age category (years), n (%)				
21-30	1 (1.1)	1 (0.5)	2 (0.6)	0.051
31-40	4 (4.4)	5 (2.3)	9 (2.9)	
41-50	11 (12.2)	15 (6.8)	26 (8.4)	
51-60	25 (27.8)	29 (13.1)	54 (17.4)	
61-70	24 (26.7)	75 (33.9)	99 (31.8)	
71-80	21 (23.3)	57 (25.8)	78 (25.1)	
81-90	4 (4.4)	39 (17.6)	43 (13.8)	
Diabetes mellitus	31 (34.4)	106 (48.0)	137 (44.1)	0.093
Hypertension	69 (76.7)	181 (81.9)	250 (80.4)	0.574
BMI category (kg/m²), n (%)				
<18.5	0 (0)	4 (1.8)	4 (1.3)	0.887
18.5-24.9	20 (22.2)	53 (24.0)	73 (23.5)	
25.0-29.9	39 (43.3)	82 (37.1)	121 (38.9)	
30.0-34.9	20 (22.2)	53 (24.0)	73 (23.5)	
35.0-39.9	3 (3.3)	15 (6.8)	18 (5.8)	
≥40	6 (6.7)	8 (3.6)	14 (4.5)	
Missing	2 (2.2)	6 (2.7)	8 (2.6)	
Obesity	28 (31.1)	73 (33.0)	101 (32.5)	0.948
Chronic kidney disease	3 (3.3)	11 (5.0)	14 (4.5)	0.818
Acute kidney injury	7 (7.8)	42 (19.0)	49 (15.8)	0.048
End-stage renal disease	1 (1.1)	9 (4.1)	10 (3.2)	0.406
Heart failure	1 (1.1)	4 (1.8)	5 (1.6)	0.906
COPD	0 (0)	5 (2.3)	5 (1.6)	0.355
Thrombocytopenia	4 (4.4)	16 (7.2)	20 (6.4)	0.660
Anemia	10 (11.1)	59 (26.7)	69 (22.2)	0.011
Coronary artery disease	21 (23.3)	82 (37.1)	103 (33.1)	0.065

Bleeding outcomes varied significantly depending on the access site (p=0.0179). Minor bleeding was detected in one patient (1.1%) in the radial group and in 21 patients (9.5%) in the femoral group. No cases of major bleeding were registered in either group (Figure 2).

**Figure 2:**
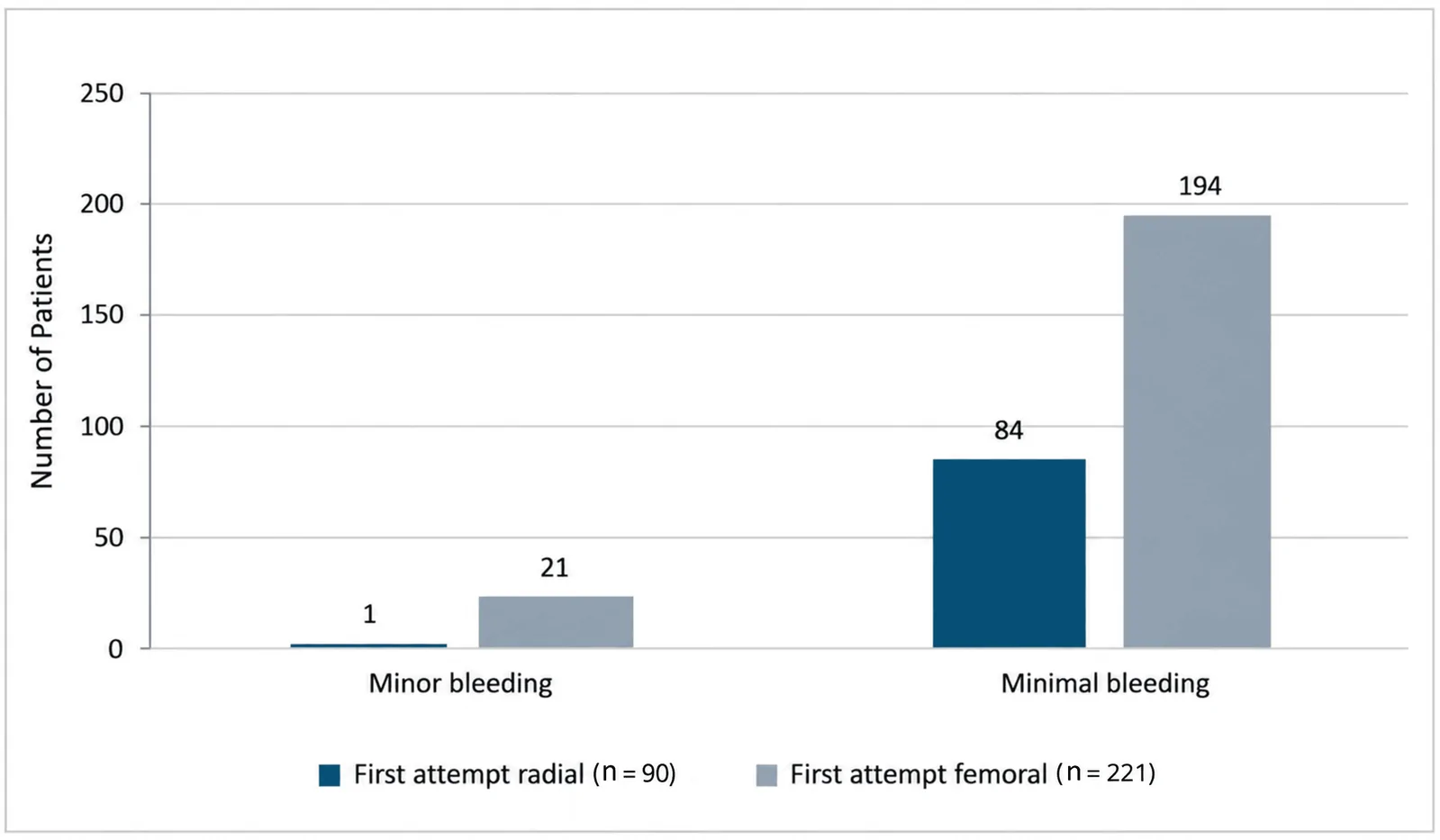
Bleeding classification according to arterial access site in patients undergoing PCI (N=311). Minor bleeding occurred in one patient (1.1%) in the radial access group and 21 patients (9.5%) in the femoral access group. No major bleeding events were reported in either group. Bleeding outcomes differed significantly according to arterial access site (p=0.0179). Statistical analysis was performed using Pearson's chi-square test (χ²=5.21). PCI: percutaneous coronary intervention

Pseudoaneurysm was not observed in the radial access group (0/90, 0%) and occurred in seven patients in the femoral access group (7/217, 3.2% among patients with available data). Four patients in the femoral group had missing pseudoaneurysm data (Figure 3). Given the small number of events and the zero cell count in the radial group, we used Fisher’s exact test for this comparison. The difference between groups was not statistically significant (p=0.1103) (Figure 4).

**Figure 3:**
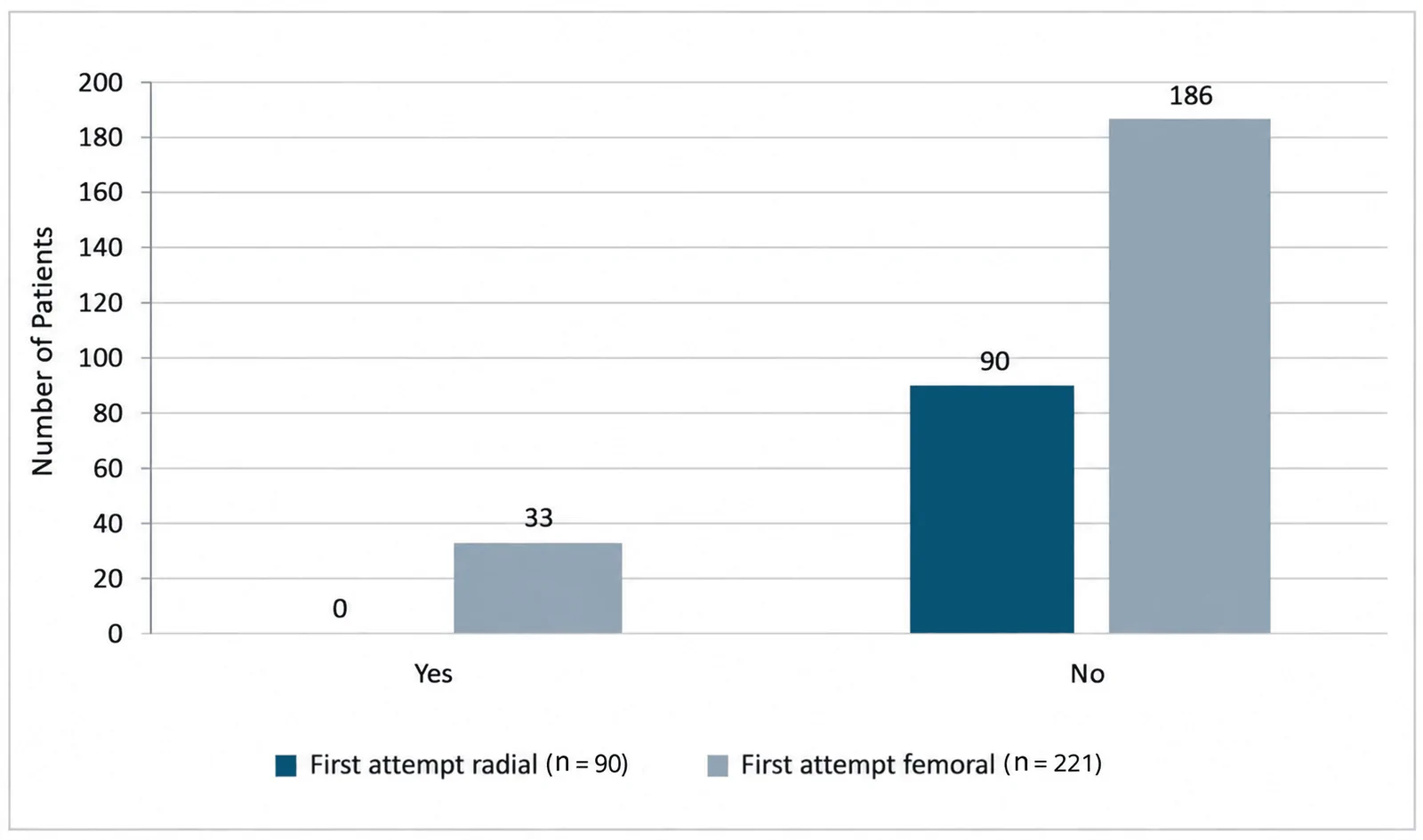
Access site hematoma according to arterial access site (N=311). Hematoma occurrence differed significantly between the radial and femoral access groups (χ²=13.645, p<0.001). Statistical analysis was performed using Pearson's chi-square test.

**Figure 4:**
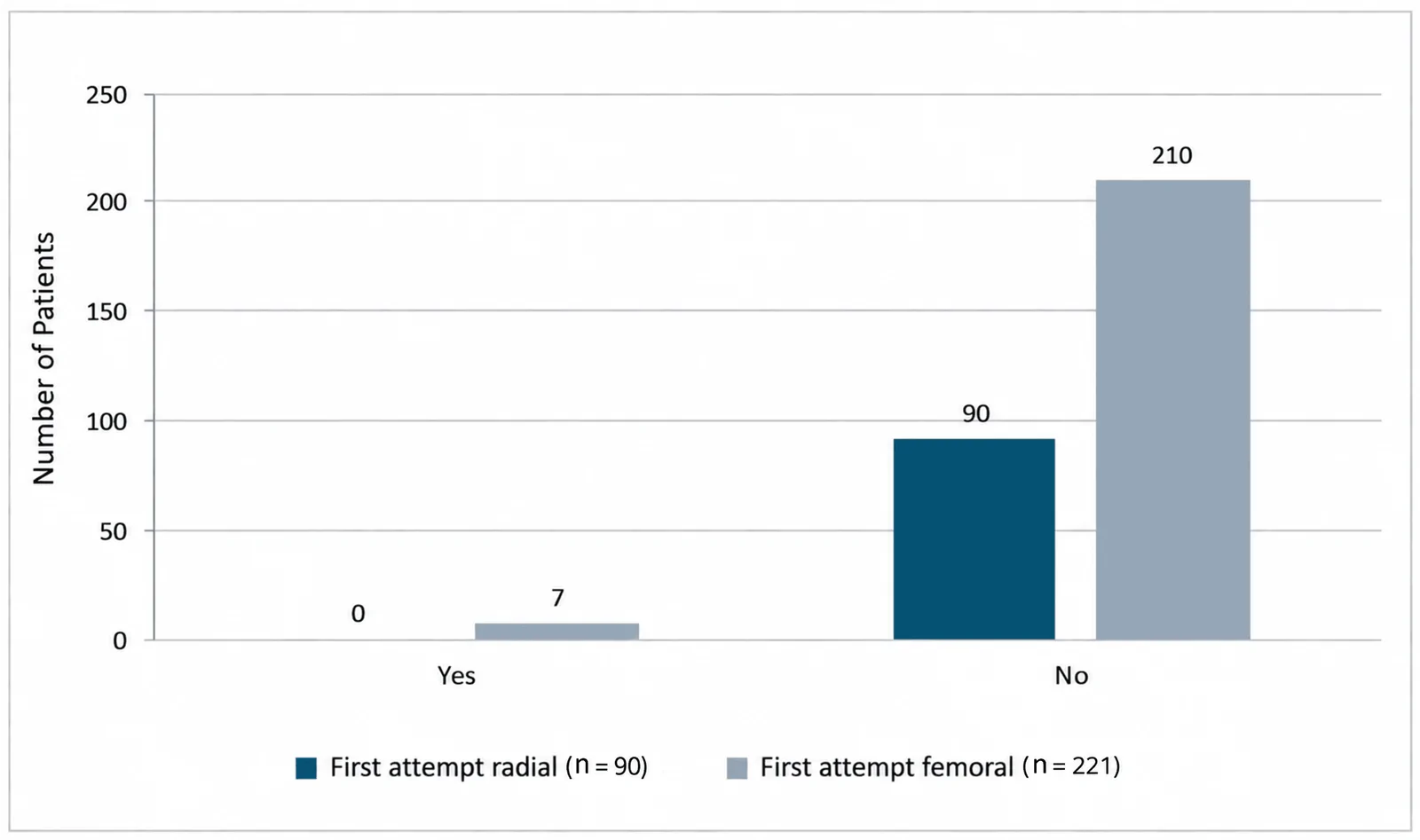
Comparison of pseudoaneurysm occurrence between radial and femoral access. Pseudoaneurysm occurred in seven patients (3.2%) in the femoral access group and was not observed in the radial access group. Four patients in the femoral group had missing data for this outcome. The difference between groups was not statistically significant (Fisher’s exact test, p=0.1103).

Procedure duration was significantly longer in the radial access group compared with the femoral access group (37.23±17.36 min versus 32.46±21.87 min, respectively), with a mean difference of 4.77 min (Welch’s t-test: t=2.03, df=206.35, 95% CI: 0.14-9.40; p=0.043) (Table 2).

**Table 2:** Comparison of procedure duration between radial and femoral access groups. Values are presented as mean±standard deviation (SD). Differences in procedure duration between radial and femoral access groups were assessed using Welch’s two-sample t-test. Mean difference, t-statistic, degrees of freedom (df), 95% confidence interval (CI), and p-value are reported. A p<0.05 was considered statistically significant.

Variables	Radial access (n=90), mean±SD	Femoral access (n=221), mean±SD	Mean difference	t-statistic	df	95% CI	p-Value
Procedure duration (min)	37.23±17.36	32.46±21.87	4.77	2.03	206.35	0.14-9.40	0.043

Thirty-day all-cause mortality occurred in one of 90 patients (1.1%) in the radial group and 26 of 221 patients (11.8%) in the femoral group. Fisher’s exact test demonstrated a statistically significant difference in 30-day mortality between the groups (p=0.0012) (Figure 5). The odds of 30-day all-cause mortality were higher in the femoral group compared with the radial group (OR: 11.81; 95% CI: 1.88-490.86).

**Figure 5:**
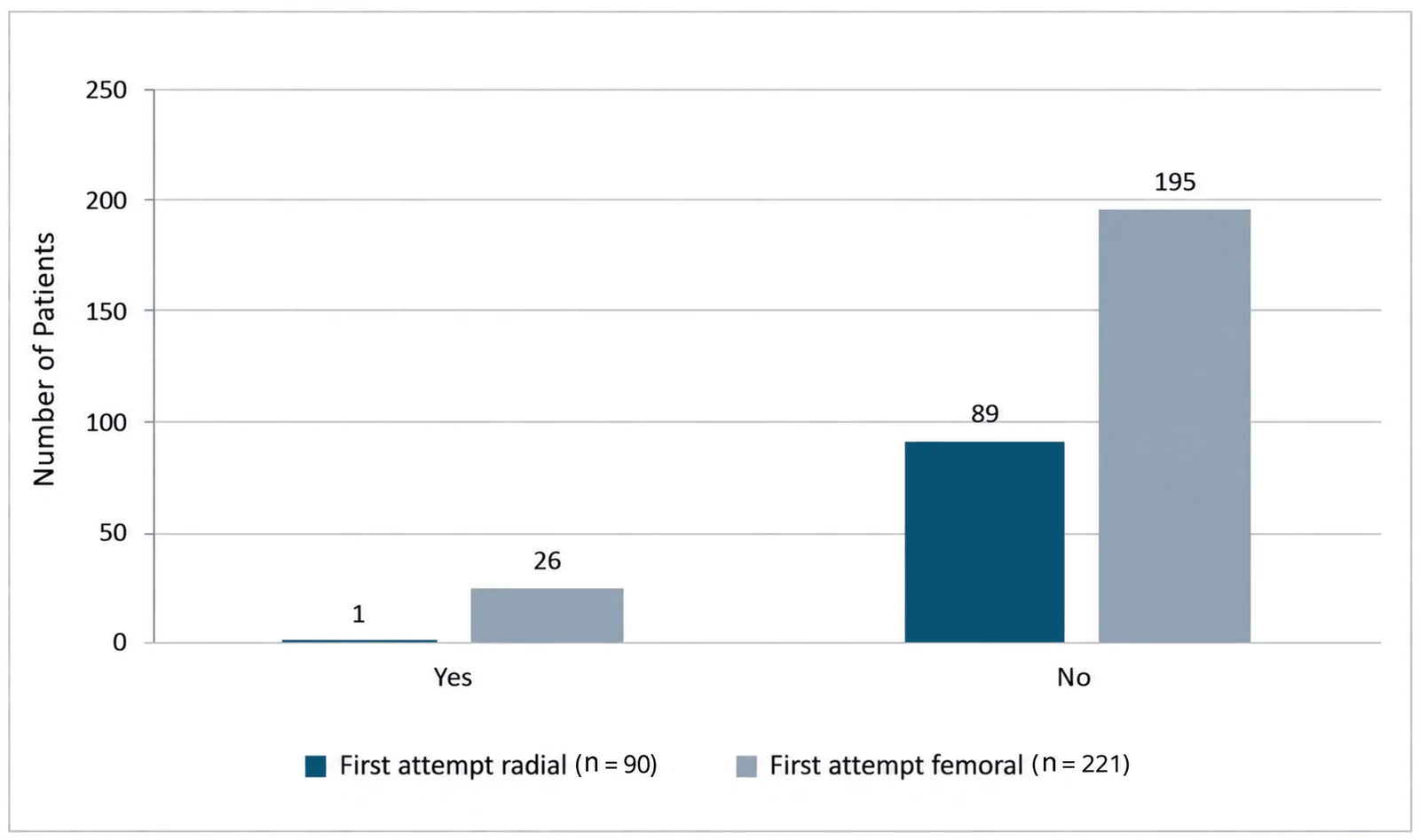
Comparison of mortality between radial and femoral access. Thirty-day all-cause mortality occurred in one of 90 patients (1.1%) in the radial access group and 26 of 221 patients (11.8%) in the femoral access group. The difference was statistically significant (Fisher’s exact test, p=0.0012). The odds of 30-day mortality were higher in the femoral group compared with the radial group (OR: 11.81; 95% CI: 1.88-490.86).

## Discussion

In this cohort study of patients with ST-segment elevation myocardial infarction (STEMI) undergoing percutaneous coronary intervention (PCI) in southern Puerto Rico, radial access was associated with fewer vascular and bleeding-related complications than femoral access. Hematoma occurred in 0% of patients undergoing radial access compared with 14.9% of those undergoing femoral access (p<0.001), while post-PCI blood transfusion was required in 3.3% and 13.1% of patients, respectively (p=0.036). Major complications were also less frequent in the radial group (11.1% versus 29.4%, p=0.003). These findings are consistent with previous randomized trials and a meta-analysis reporting lower rates of major bleeding and vascular complications with radial access [2-4,6-8,10,11]. Contemporary clinical guidelines similarly recommend radial access as the preferred approach in patients with acute coronary syndrome (ACS) undergoing PCI to reduce bleeding, vascular complications, and mortality [1].

Our findings are consistent with the Radial Versus Femoral Randomized Investigation in ST-Elevation Acute Coronary Syndrome (RIFLE-STEACS) trial, in which radial access was associated with lower rates of net adverse clinical events (13.6% versus 21.0%), bleeding (7.8% versus 12.2%), and cardiac mortality (5.2% versus 9.2%) compared with femoral access [2]. Similarly, previous evidence focusing on patients undergoing primary PCI for STEMI has demonstrated reductions in mortality, major bleeding, and access-site bleeding with radial access [4]. A systematic review and meta-analysis of randomized trials also demonstrated reductions in major bleeding, vascular complications, and mortality with radial compared with femoral access [6]. These findings support the potential clinical benefit of minimizing access-site complications in patients undergoing PCI for acute myocardial infarction.

Additional evidence from the MATRIX program further supports the use of radial access in patients with ACS. MATRIX randomized 8,404 patients to radial or femoral access and demonstrated fewer net adverse clinical events with radial access at one year (15.2% versus 17.2%; rate ratio: 0.87; 95% CI: 0.78-0.97), although the difference in major adverse cardiovascular events at one year did not reach statistical significance [10]. These findings reinforce the potential safety advantage of radial access while also illustrating that the magnitude of benefit may differ according to the clinical outcome examined.

Thirty-day all-cause mortality in our cohort was 1.1% in the radial group compared with 11.8% in the femoral group (p=0.0012). Although this is consistent with meta-analyses demonstrating a benefit in terms of mortality with radial access, the magnitude of the mortality difference observed in the present study should be interpreted cautiously [4,6,8,10,11]. The mortality odds ratio was 11.81; however, the very wide 95% confidence interval (1.88-490.86) indicates substantial statistical imprecision, likely driven by the small number of mortality events, particularly the single death observed in the radial group. Therefore, the point estimate should not be interpreted as a precise measure of the magnitude of the mortality difference between access strategies. In contrast to studies demonstrating a mortality advantage, the SAFARI-STEMI randomized clinical trial did not demonstrate a significant difference in 30-day mortality between radial and femoral access [5]. The contemporary ACC/AHA ACS guideline also notes that SAFARI-STEMI was terminated early and was therefore underpowered to detect a mortality benefit. Although the difference in procedure duration reached statistical significance, the absolute mean difference was only 4.77 min. The clinical relevance of this modest difference in the context of STEMI PCI is uncertain and is unlikely, by itself, to represent a major disadvantage of radial access. Therefore, this finding should be interpreted cautiously, particularly given the retrospective design and the potential influence of procedural complexity and operator-related factors.

Importantly, the observed mortality difference in this cohort should not be interpreted as demonstrating a causal effect of vascular access. Patients undergoing femoral access had higher rates of anemia (26.7% versus 11.1%, p=0.011) and acute kidney injury (19.0% versus 7.8%, p=0.048), suggesting that baseline clinical differences and preferential selection of femoral access in higher-risk patients may have contributed to the mortality difference. Because vascular access was not randomized and these potential confounders were not controlled through multivariable adjustment, the association between femoral access and mortality may partially reflect differences in baseline risk rather than the vascular access strategy alone.

The observed reduction in bleeding and vascular complications with radial access is clinically relevant because bleeding complications after PCI have been associated with adverse outcomes, and reducing access-site bleeding represents an important component of contemporary PCI practice. The 2025 American College of Cardiology (ACC)/American Heart Association (AHA)/American College of Emergency Physicians (ACEP)/National Association of EMS Physicians (NAEMSP)/Society for Cardiovascular Angiography and Interventions (SCAI) guideline recommends a radial approach over a femoral approach in patients with ACS undergoing PCI to reduce bleeding, vascular complications, and mortality (Class 1, Level of Evidence A) [1]. The guideline evidence review also reports that individual-patient data from seven randomized trials demonstrated relative reductions in all-cause mortality, major bleeding, and vascular complications with radial access.

The present study adds data from a Puerto Rican population, which has been less represented in major randomized trials evaluating PCI vascular access strategies. Although our observational results cannot establish causality, the consistency of the bleeding and vascular findings with larger randomized studies supports the applicability of a radial-first strategy in appropriately selected patients with STEMI treated in southern Puerto Rico. Nevertheless, femoral access remains clinically necessary in selected circumstances, including patients in whom radial access is technically or anatomically unsuitable or when large-bore access or mechanical circulatory support is required. Contemporary guidelines similarly recognize femoral access as an important alternative when radial access cannot be used for clinical, anatomical, or technical reasons [1].

Limitations

This study has several limitations. First, its retrospective, single-center design is subject to the inherent limitations of medical record review and may limit the generalizability of the findings. Second, the selection of radial or femoral access was not randomized and may have been influenced by patient characteristics, clinical presentation, procedural complexity, and operator preference, introducing the possibility of selection bias and residual confounding. In addition, cardiogenic shock, hemodynamic instability, and the use of mechanical circulatory support were not systematically captured in the study dataset. These factors may have influenced vascular access selection and clinical outcomes, particularly if femoral access was preferentially used in higher-risk or unstable patients, and therefore represent potential sources of unmeasured confounding. This concern is particularly relevant given the higher prevalence of anemia and acute kidney injury in the femoral group.

Furthermore, the limited number of 30-day mortality events, including only one death in the radial group, precluded a reliable conventional multivariable analysis incorporating multiple potential confounders because of the risk of model overfitting and unstable estimates. Consequently, residual confounding cannot be excluded, and the observed mortality association should not be interpreted as independent or causal. The small number of mortality events also resulted in an extremely wide confidence interval around the mortality odds ratio (OR: 11.81; 95% CI: 1.88-490.86), indicating substantial statistical imprecision and limiting interpretation of the magnitude of the observed association.

Additionally, the unequal sample sizes between the radial and femoral groups may have affected the precision of between-group comparisons. Incomplete documentation affected 11.9% of patients for at least one study variable, although missingness for individual variables was generally low. Missing observations were not uniformly distributed between access groups, with most missing procedural and outcome data occurring in the femoral group; therefore, the potential for bias related to missing data cannot be excluded. The small number of pseudoaneurysm events similarly limited statistical precision for that outcome. Finally, because the study was conducted at a single hospital serving southern Puerto Rico, the findings may not be representative of other institutions or populations. Larger prospective, multicenter studies with a greater number of events and adequate adjustment for potential confounders are warranted to confirm these findings.

## Conclusions

In this single-center retrospective study of patients with STEMI undergoing PCI in southern Puerto Rico, radial access was associated with lower rates of bleeding and vascular complications compared with femoral access. Radial access was also associated with lower observed 30-day all-cause mortality; however, this finding should be interpreted cautiously given the non-randomized study design, baseline differences between groups, the small number of mortality events, and the lack of multivariable adjustment. These findings demonstrate associations rather than causal effects of vascular access strategy. Larger prospective, multicenter studies with adequate adjustment for potential confounders are needed to further evaluate these associations in Puerto Rican patients with STEMI.
